# Receptivity and preferences of pancreatic cancer family members for participating in lifestyle programs to reduce cancer risk

**DOI:** 10.1186/1897-4287-11-3

**Published:** 2013-05-31

**Authors:** Lisa A Howell, Pamela S Sinicrope, Tabetha A Brockman, Christi A Patten, Paul A Decker, Shawna L Ehlers, Ashley Nadeau, Kari G Rabe, Carmen Radecki Breitkopf, Gloria M Petersen

**Affiliations:** 1 Department of Psychology and Psychiatry, Mayo Clinic Rochester, Rochester, MN, USA; 2 Behavioral Health Research Program, Mayo Clinic Rochester, Rochester, MN, USA; 3 Division of Biomedical Statistics and Informatics, Department of Health Sciences Research, Mayo Clinic Rochester, Rochester, MN, USA; 4 Division of Health Care Policy & Research, Department of Health Sciences Research, Mayo Clinic Rochester, Rochester, MN, USA; 5 Division of Epidemiology, Department of Health Sciences Research, Mayo Clinic Rochester, Rochester, MN, USA

**Keywords:** Pancreatic cancer, Family, Health behavior change, Perceived risk, Risk reduction, Lifestyle

## Abstract

**Background:**

Cancer is a shared family experience that might provide an opportunity for lifestyle change among at-risk family members. The purpose of this study was to assess receptivity and preferences for cancer risk reduction programs among at-risk family members with two or more relatives affected with pancreas cancer.

**Methods:**

We surveyed 401 at-risk family members in an existing pancreatic cancer family registry. Participants completed a mailed survey which examined demographic, medical, and psychosocial correlates of willingness to participate in lifestyle cancer risk reduction programs. Multivariable generalized estimating equation approaches were used to model preferences.

**Results:**

Overall, 85% (n = 342) of at-risk family members were receptive to lifestyle cancer risk reduction programs. Participant preferred programs focused on nutrition (36%, n = 116) and weight management (33%, n = 108), with Web/Internet (46%, n = 157) being the most preferred delivery channel. Most respondents preferred to participate in programs with their family or friends (74%, n = 182), rather than alone (25%, n = 85). In multivariable analysis, younger age (p = 0.008) and higher perceived likelihood of developing cancer (p = 0.03) were associated with willingness to participate in lifestyle programs.

**Conclusions:**

Family members of those with pancreatic cancer are receptive to cancer risk reduction programs focusing on nutrition and weight management delivered via the internet. Further research is indicated to determine how to best incorporate a family-based approach when designing lifestyle intervention programs.

## Introduction

Cancer is a shared family experience and awareness of an increased cancer risk due to family history may provide an opportunity for cancer risk reduction behaviors among at-risk but unaffected family members. With this in mind, it may be logical to approach behavior change efforts at the familial level. This situation is especially salient in the case of pancreatic cancer (PC), given its poor prognosis, limited means of treatment and diagnosis, and lack of firm information about PC prevention [1-4].

Pancreatic cancer is the second most common form of digestive tract cancer and the fourth leading cause of cancer death [2,5], with an estimated 6% of patients surviving 5 years post diagnosis. Low survival rates are due to the fact that in early stages PC is largely asymptomatic, and early detection is limited [2]. The only surveillance techniques currently used to facilitate diagnosis of PC are computed tomography, magnetic resonance imaging, and endoscopic ultrasound, all of which cannot feasibly be applied to widespread use, and are not proven to reduce PC morbidity and mortality [6]. As a result, PC is one of the few cancers with little improvement in survival rates over the past 40 years.

Lifestyle risk factors, including tobacco use, physical inactivity, diet high in red meat and fat, and higher body mass index (BMI) are related to the development of PC [2,5,7-10]. Moreover, having at least one first degree relative with PC significantly increases risk as much as 2 to 5 times that of someone with no family history, and the risk is even higher for those with more than one affected relative [2,5,11,12].

A cancer diagnosis has been found to be a teachable moment for health behavior change (e.g., smoking cessation) among those diagnosed [13-15]. However, the timing of receptivity to behavior change in relation to the patient’s cancer diagnosis is not well understood for family members. It is possible that family members may want to make lifestyle changes after some time has passed post-diagnosis, given intense emotional reactions, fatigue related to caregiving, or concern about their own cancer risk.

While a significant amount of research has explored how caregiving duties, coping, and psychosocial distress [16] can have an impact on families, there is a paucity of research exploring how the cancer experience might influence cancer-prevention behavior change among family members. Moreover, few studies have developed and/or explored the use of family-based interventions for those diagnosed with cancer and their family members, and none currently explored this issue with respect to PC. A recent study found that 54% of cancer patients were interested in helping a friend or family member stop smoking in an effort to decrease their relative’s cancer risk [17]. Kristeller and colleagues (1996) [11] found that family members were receptive to discussing cancer and possible interventions, but that spontaneous behavior change was low among family members. Thus, it is unclear if family members initiate and make actual changes in their behavior to reduce cancer risk, even if they are receptive and willing to make changes.

Several questions remain with respect to preferences among family members for types of interventions (exercise, weight management), program format (family versus individual), and/or delivery channels (web-based versus face-to-face). Also, investigations into aspects of the family cancer experience (e.g., timing of diagnosis to program recruitment) are needed.

To address these gaps, our overall study objectives were two-fold: 1) assess receptivity and preferences for participating in cancer risk reduction programs among PC family members and 2) to identify demographic, medical, psychosocial, and behavioral characteristics associated with willingness to participate in a risk reduction intervention. These data were collected as the first step toward developing lifestyle interventions that incorporate the family cancer experience.

## Methods

The study was approved by the Mayo Clinic Institutional Review Board.

### Participants and procedures

Participants included at-risk unaffected family members of patients (probands) who were diagnosed and/or treated for PC at Mayo Clinic [18]. The probands were recruited into a PC patient research registry established in 2001, and had provided detailed risk factor, clinical, and family history information. Currently, the PC family registry consists of 1570 consented and living adult relatives in 264 families (212 spouses and 1358 blood relatives). Eligibility for at-risk unaffected family members included a positive family history (two or more relatives affected with PC), age 18 or older, and ability to provide written informed consent.

In March 2009, 461 subjects in the PC family registry were mailed a packet that included: 1) a cover letter explaining the purpose of the research; 2) a consent form; 3) a survey (described below) with a postage-paid, pre-addressed return envelope. A second packet was mailed to those who did not respond within approximately 6 weeks of the first mailing. No remuneration was offered for participation. Data were available for 401 (87%) participants who are the focus of this report.

### Measures

Demographic and medical data were furnished by the family registry database and abstracted from medical records, including age, gender, race, marital status, education level, body mass index, smoking status, number of first degree relatives with PC, and time since PC diagnosis of the affected family member(s).

The survey was developed to address the content areas described below and then pilot-tested on 22 adults with cancer family history of PC and then revised accordingly. Psychosocial characteristics measured were perceived and comparative cancer risk (for PC and cancer generally) [4] degree of cancer worry/concern (for PC and cancer generally) [4]; degree of emotional closeness to affected family member(s) [19]; and self-efficacy for behavioral change in general [20] as well as for nutrition [21] and exercise [22].

Health behaviors assessed from the survey were physical activity (PA); Godin Leisure Time Exercise Questionnaire (GLTEQ) [23], dietary behavior over the past month [21], alcohol consumption over the past year, and smoking status obtained from the medical records.

Receptivity to participate in a cancer risk reduction lifestyle program was measured by the question, “How willing would you be to take part in a lifestyle program (i.e., exercise, nutrition, smoking cessation) to help reduce your risk of getting cancer if we were to create one?” Response categories included, “Not at all”, “Somewhat”, and “Definitely”. Those responding “Somewhat” or “Definitely” were asked five additional questions to assess program preferences. The first question was “Would you want a program that was just for you or one that includes you and your family or others?” Categories of response were “Just me”, “Me and my family”, and “Me and others (i.e., people outside family like friends or coworkers)”. The second question was “What type of lifestyle program(s) would be of interest?” with instructions to “Mark ALL that apply”. Choices included: Exercise, Weight management, Nutrition, Tobacco cessation (to quit smoking), Stress reduction, and Other (specify). The third question asked subjects to select their top choice from the types of lifestyle program(s) listed above. The fourth question was “How likely would you be to take part if the program was delivered by phone, by mail, in person, by internet?” Categories of response were on a 3-point Likert scale ranging from “Very likely” to “Not at all likely”. The fifth question invited participants to select their top choice out of the four ways to deliver the program.

### Statistical analysis

Predictors of receptivity to participate (“Definitely” or “Somewhat” willing versus “Not” willing) in a health promoting lifestyle program among family members was assessed with generalized estimating equations to account for multiple family members who might participate from a given kindred. Both univariable and multivariable models were fit. Variables that were significant in univariable analyses were included in the multivariable model. In all cases, p-values <0.05 were considered statistically significant. Descriptive data are presented as frequencies (n), percentages, means, and standard deviations (SD).

## Results

### Respondent characteristics

Table 1 displays the characteristics of the 401 respondents. The respondents were middle aged (mean 58.5 years, range 23–96; SD = 13.4), Caucasian (98.5), mostly female (63%), highly educated (82% with more than 12 years of education), and 78% (n = 240) reported being married or having a life partner. The mean BMI of respondents was 27.9 kg/m^2^ (range 17.4-57.5; SD = 5.3) with 69% meeting criteria for being overweight or obese (BMI above 25). About half (51%) of the respondents reported engaging in moderate to strenuous physical activity (Godin score > 24), and most reported being in good to excellent health (88%). Respondents endorsed eating moderately healthy diets. Approximately half (48%) of indicated consuming alcohol weekly and 11% were current smokers. All respondents had one or more family members affected by PC. The time since diagnosis of the most recently affected family member was 7.2 years (range: 0–33 years; SD = 7.5).

**Table 1:** Pancreatic cancer family member respondent characteristics (N = 401)

	**# (%)**
**Age**	
N	395
Mean (SD)	58.5 (13.4)
Range	(23.0-96.0)
**Gender**	
Female	251 (62.6%)
Male	150 (37.4%)
**Caucasian**	
No	6 (1.5%)
Yes	384 (98.5%)
Missing	11
**Married/life partner**	
No	69 (22.3%)
Yes	240 (77.7%)
Missing	92
**Education level**	
Elementary school or junior high	6 (1.5%)
High school/GED	65 (16.4%)
Some college/trade school	125 (31.5%)
College degree	113 (28.5%)
Postgraduate degree	88 (22.2%)
Missing	4
**Body Mass Index (kg/m**^**2**^**)**	
N	392
Mean (SD)	27.9 (5.3)
Median	27.2
Range	(17.4-57.5)
<25	123 (31.4%)
25-30	151 (38.5%)
>30	118 (30.1%)
**Godin Score for physical activity**	
N	332
Mean (SD)	30.6 (30.2)
Range	(0.0-250.0)
Score < = 24	191 (49.4%)
Score > 24	196 (50.6%)
**Diet Score**	
N	397
Mean (SD)	3.3(0.6)
Range	(1.5-4.6)
**On average, how many drinks of alcohol do you usually have?**	
None	34 (8.8%)
Less than one each month	83 (21.5%)
1 to 3 each month	77 (19.9%)
1 to 2 each week	56 (14.5%)
3 to 6 each week	69 (17.9%)
1 to 2 each day	52 (13.5%)
3 or more each day	15 (3.9%)
Missing	15
**Smoking cigarettes**	
Never	191 (47.6%)
Former smoker	157 (39.1%)
Current smoker	44 (11.0%
Missing	9
**Number of first degree relatives with pancreas cancer**	
0-1	352 (87.8%)
2	45 (11.2%)
3 or more	4 (0.9%)
**Time from pancreas cancer diagnosis of proband to survey completion by relative (years)**	
N	375
Mean (SD)	7.1 (7.3)
Median	4.7
Q1, Q3	2.6, 7.7
Range	(0.1-33.1)

### Receptivity to participate in a lifestyle intervention and program preferences

Table 2 shows self-reported willingness of respondents to participate in cancer risk reduction lifestyle interventions. The majority of respondents (85.3%, n = 342) were “Somewhat” or “Definitely” willing to participate in a lifestyle cancer-reduction program. Among those receptive to the programs, about half (54%) preferred to engage in a program with other family members. The most preferred programs included nutritional information (36%) and weight management (34%), with the Web/Internet being the preferred delivery channel (46%).

**Table 2:** Cancer risk-reduction program receptivity among pancreatic cancer family members (N = 401)

	**# (%)**
**How willing would you be to take part in a lifestyle program (i.e., exercise, nutrition, smoking cessation) to help reduce your risk of getting cancer if we were to create one?**	
Not at all	59 (14.7%)
Somewhat	167 (41.6%)
Definitely	175 (43.6%)
Missing	0
**If you answered somewhat or definitely, would you want a program that was just for you or one that includes you and your family or others?**	
Just me	85 (25.4%)
Me and my family	182 (54.3%)
Me and others (i.e., friends or coworkers)	68 (20.3%)
Missing	7
**Which of the programs listed above would be your top choice?**	
Exercise	49 (15.4%)
Weight management	108 (33.9%)
Nutrition	116 (36.4%)
Tobacco cessation	15 (4.7%)
Stress reduction	31 (9.7%)
Missing	23
**Of the four ways to deliver the program, which one would be your top choice?**	
Telephone	9 (2.7%)
Web/Internet	157 (46.3%)
In person	80 (23.6%)
Mail	93 (27.4%)
Missing	3

### Correlates of receptivity to participate in a lifestyle intervention

Family members who reported receptivity to lifestyle interventions (relative to those who were not receptive) were generally younger (p < 0.001), female (p = 0.01), and more educated (p = 0.012) (Table 3). Those willing to participate in risk reduction interventions were higher in subjective general self efficacy (p = 0.03), nutritional self efficacy (p = 0.02), and in exercise self efficacy (p =0.01) compared to those who were not willing. In addition, those willing to participate perceived themselves as having a greater risk of developing PC themselves (p = 0.0007), and perceived greater risk of developing other forms of cancer (p = 0.0009). Similarly, this group reported higher levels of concern about getting PC (p < 0.0001) and higher levels of concern about cancer in general (p = 0.001).

**Table 3:** Comparison of demographic, psychosocial and health behavior characteristics by pancreatic cancer family members receptiveness to participate in lifestyle interventions (N = 401)

	**Not willing (N = 59)**	**Somewhat or definitely willing (N = 342)**	**p value†**
**Age**			<0.0001
N	59	336	
Mean (SD)	68.3 (14.9)	56.8 (12.4)	
Range	(23.0-96.0)	(24.0-92.0)	
**Gender**			0.01
Female	26 (44.1%)	225 (65.8%)	
Male	33 (55.9%)	117 (34.2%)	
Caucasian			0.88
No	1 (16.7%)	5 (83.3%)	
Yes	55 (14.3%)	329 (85.7%)	
Missing	3	8	
Married/life partner			0.29
No	13 (18.8%)	56 (81.2%)	
Yes	32 (13.3%)	208 (86.7%)	
Missing	14	78	
**Education level**^**a**^			0.012
Elementary school or junior high	2 (3.5%)	4 (1.2%)	
High school/GED	17 (29.3%)	48 (14.2%)	
Some college/trade school	19 (32.8%)	106 (31.3%)	
College degree	11 (19.0%)	102 (30.1%)	
Postgraduate degree	9 (15.5%)	79 (23.3%)	
Missing	1	3	
**Body Mass Index (kg/m**^**2**^**)**			0.13
N	57	335	
Mean (SD)	27.0 (5.0)	28.0 (5.3)	
Median	25.8	27.4	
Q1, Q3	23.7, 29.9	24.3, 31.2	
Range	(17.4-38.8)	(17.8-57.5)	
<25	24 (19.5%)	99 (80.5%)	
25-30	19 (12.6%)	132 (87.4%)	
>30	14 (11.9%)	104 (88.1%)	
**Godin Score for physical activity**			0.31
N	55	332	
Mean (SD)	27.1 (22.6)	30.6 (30.2)	
Range	(0.0-120.0)	(0.0-250.0)	
Score < = 24	28 (14.7%)	163 (85.3%)	
Score > 24	27 (13.8%)	169 (86.2%)	
**Diet Score**			0.48
N	56	341	
Mean (SD)	3.3 (0.6)	3.3 (0.5)	
Range	(1.5-4.6)	(2.1-4.6)	
**Average drinks of alcohol**			0.19*
None	9 (16.7%)	25 (7.5%)	
Less than one each month	10 (18.5%)	73 (22.0%)	
1 to 3 each month	5 (9.3%)	72 (21.7%)	
1 to 2 each week	10 (18.5%)	46 (13.9%)	
3 to 6 each week	11 (20.4%)	58 (17.5%)	
1 to 2 each day	7 (13.0%)	45 (13.6%)	
3 or more each day	2 (3.7%)	13 (3.9%)	
Missing	5	10	
**Smoke cigarettes**			0.31
Never	23 (39.7%)	168 (50.3%)	
Former smoker	27 (46.6%)	130 (38.9%)	
Current Smoker	8 (13.8%)	36 (10.8%)	
Missing	1	8	
**Number of first degree relatives with pancreas cancer**			0.57
0-1	49 (83.1%)	303 (88.6%)	
2	9 (15.3%)	36 (10.5%)	
3 or more	1 (1.7%)	3 (0.9%)	
**Time from pancreas cancer diagnosis of proband to survey completion by relative (years)**			0.48
N	54	321	
Mean (SD)	7.9 (8.3)	7.1 (7.3)	
Median	5.6	4.7	
Q1, Q3	2.7, 7.6	2.6, 7.7	
Range	(0.1-32.3)	(0.1-33.1)	
**General self-efficacy score**			0.03
N	58	339	
Mean (SD)	27.2 (4.3)	28.6 (3.8)	
Range	(17.0-36.0)	(12.0-36.0)	
**Nutrition self-efficacy score**			0.02
N	58	339	
Mean (SD)	13.8 (3.5)	15.1 (3.2)	
Range	(5.0-20.0)	(5.0-20.0)	
**Exercise self-efficacy score**			0.01
N	57	331	
Mean (SD)	24.0 (11.0)	27.9 (10.4)	
Range	(5.0-50.0)	(5.0-50.0)	
**Overall health:**			
**Compared to other people your age, how would you describe your state of health?**			0.06**
Excellent	10 (17.5%)	51 (15.0%)	
Very good	18 (31.6%)	140 (41.1%)	
Good	14 (24.6%)	116 (34.0%)	
Fair	13 (22.8%)	27 (7.9%)	
Poor	2 (3.5%)	7 (2.1%)	
Missing	2	1	
**Cancer risk:**			
**How likely do you think it is that you will get pancreas cancer?**			0.0007
Very likely	2 (3.4%)	19 (5.6%)	
Somewhat likely	12 (20.3%)	149 (44.0%)	
Somewhat unlikely	17 (28.8%)	88 (26.0%)	
Very unlikely	14 (23.7%)	32 (9.4%)	
I have no feeling or opinion on my chances of getting PC	14 (23.7%)	51 (15.0%)	
Missing	0	3	
**How likely do you think it is that you will get cancer?**			0.0009
Very likely	2 (4.8%)	41 (14.4%)	
Somewhat likely	13 (31.0%)	138 (48.6%)	
Somewhat unlikely	5 (11.9%)	55 (19.4%)	
Very unlikely	9 (21.4%)	15 (5.3%)	
I have no feeling or opinion on my chances of getting cancer	13 (31.0%)	35 (12.3%)	
Missing	17	58	
**Concern:**			
**How concerned are you about getting pancreas cancer?**			<0.0001
Extremely concerned	2 (3.4%)	54 (15.8%)	
Moderately concerned	4 (6.9%)	92 (27.0%)	
Mildly concerned	23 (39.7%)	143 (41.9%)	
Not at all concerned	29 (50.0%)	52 (15.2%)	
Missing	1	1	
**How concerned are you about getting cancer?**			0.001
Extremely concerned	2 (4.5%)	30 (10.6%)	
Moderately concerned	5 (11.4%)	92 (32.5%)	
Mildly Concerned	22 (50.0%)	138 (48.8%)	
Not at all concerned	15 (34.1%)	23 (8.1%)	
Missing	15	59	
**Emotional closeness:**			
**How close is (or was) your relationship with the family member diagnosed with pancreas cancer?**			0.34‡
Closer than any relationship I’ve had before or since	15 (30.6%)	78 (24.5%)	
Closer than most relationships I’ve had with other people	14 (28.6%)	138 (43.3%)	
About as close as most relationships with others	15 (30.6%)	65 (20.4%)	
Not as close as most relationships	3 (6.1%)	22 (6.9%)	
Not very close at all	2 (4.1%)	16 (5.0%)	
Missing	10	23	
**Caregiver:**			
**Have you ever been directly involved as a caregiver for a loved one with cancer?**			0.24
Yes	31 (53.4%)	211 (62.2%)	
No	27 (46.6%)	128 (37.8%)	
Missing	1	3	

† Adjusted P-value from Generalized Estimating Equations model for related family members.

^a^ Comparing subjects with some college education versus others.

* P-value obtained is from combining 1 to 2 drinks each day and 3 or more drinks each day.

** P-value obtained from combining fair and poor.

‡ P-value obtained is from combining responses “Closer than any relationship I’ve had before” and “Closer than most relationships I’ve had with other people” versus “About as close as most relationships with others” versus “Not as close as most relationships” and “Not very close at all”.

The following variables were included in the multivariable model of receptivity versus not: age, gender, education, likelihood of developing cancer, concern about getting cancer, and general self-efficacy. In this model, only younger age (p = 0.008) was associated with receptivity to participate in cancer-risk reduction programs. Trend-level associations with receptivity to participate in a cancer-risk reduction program, included higher likelihood of developing cancer (p = 0.058), higher concern of developing cancer (p = 0.060).

## Discussion

This study assessed the receptivity and preferences of at-risk family members from a PC family registry to participate in cancer risk reduction programs. Concurrently, we also assessed health behaviors of the participants as well as potential psychosocial correlates of such receptivity. We found that 85% of participants were at least “somewhat” receptive to lifestyle programs, with 34% preferring a program focusing on weight management and 37% preferring a program focusing on nutrition. These areas are related and have been shown in the literature to be effective in reducing cancer risk [7].

Notably, a majority (74%) reported a stronger preference for participating in risk reduction programs with their family members and friends. This novel finding suggests a new avenue for intervention development that incorporates natural sources of support [24]. A benefit of a natural source of support is that it can have a positive effect on one’s self-esteem which in turn can increase motivation and retention to changes in behavior. Additionally, a social network can provide coping resources such as emotional, informational, and instrumental support. At-risk family members and cancer survivors should be studied as an integrated family unit to better understand and conceptualize a family or group-based program. Future studies could use a multi-dimensional assessment tool (e.g., Cancer Risk Belief Scale [25]) to explore individuals’ ideas about the role of family in cancer risk or employ qualitative methods to achieve a more in-depth understanding.

Among respondents who were receptive to participating in risk reduction lifestyle programs, 71% meet the criteria of being overweight or obese, and 69% self-reported lower levels of physical activity, suggesting unhealthy lifestyles in this group. However, these respondents self-reported higher levels in general, nutritional, and exercise self-efficacy, reflecting that this group may be more confident in their ability to start making behavioral changes (e.g. initiate weight loss programs and nutritional information sessions). Thus, there may be tension between intentions to change versus making an actual change in lifestyle behavior, which may explain why approximately half (n = 167) of these respondents reported being “somewhat” rather than “definitely” receptive. Therefore, it is important for lifestyle programs to address barriers such as lack of motivation and retention in intervention efforts.

Our findings suggest that concern about developing cancer appears to be the largest motivating factor for willingness to participate in cancer risk reduction programs in the family members. This willingness may be due to the lethality of this cancer and the poor prognosis of those diagnosed with PC. Similarly, it can be posited that there may be more grief or impending loss concerns among the family members which may minimize their receptivity to participate in risk reduction programs. Thus, further exploration is needed to determine how to approach grief and loss concerns in future program development and recruitment efforts.

With respect to program delivery channels, family members preferred Web/Internet over other options. This preference could be due to the fact that family members in the study were geographically dispersed; and that participants’ average age was less than 50 which might suggest they are more experienced and comfortable with web-based modalities. In multivariable analysis, only younger age was associated with increased receptivity to participate in cancer risk reduction programs. This suggests that younger individuals may be more open to interventions, especially if they can anticipate benefits from the programs that decrease cancer risk.

This study found that the timing of the PC patient’s diagnosis did not affect the family member’s receptivity to participate in lifestyle interventions. However, a limitation is that family members were surveyed on average seven years after the patient was diagnosis with PC, and thus we may not have fully captured the range of possible responses based on early time since diagnosis (less than 2 years). Despite this finding, it is possible that actual participation, treatment compliance, and/or effectiveness of interventions would be different based on duration since diagnosis. Other factors such as emotional reactions, fatigue related to caregiving, or concern about their own cancer risk may also influence program participation and treatment engagement. Future studies including a shorter time since diagnosis would be necessary to accurately assess these additional factors. In the area of PC, additional work is needed to understand the impact of this cancer on family members’ perceived vulnerability and willingness to confront the potential relationships between their own behavior and their cancer risk. Similarly, it is unclear what aspects of the cancer experience should be incorporated into lifestyle programs for families affected by PC that take into account their unique needs and concerns. For instance, mental health concerns (e.g., anxiety, depression) are heightened in spouses and partners of cancer patients; the particularly poor prognosis for PC suggests that issues of mental health deserve special attention, as depression and anxiety are known to interfere with participation and success in behavior change programs [26]. In addition, the degree of connection to the family cancer experience such as emotional and cognitive involvement and perceived similarity to the affected person [27] could be assessed in subsequent work.

There is limited information about how one’s behavioral intentions may be different from actual behavior change, especially with at-risk cancer populations. There is some evidence that patient’s often feel like behavioral modification will have little impact on their prognosis, and therefore are less likely to put forth effort to make change [28]. It has been found that patient perceptions of the effectiveness of positive lifestyle changes (dietary changes, exercise, weight loss) and medical recommendations (following recommendations for screenings) are a key part in the decision-making process used to determine the extent of their medical recommendation adherence [29]. It would be valuable to explore these topics in this population to assess if intentions match actual behavioral change.

There are strengths and limitations in this study. As the purpose of this study was to explore the preferences and receptivity to lifestyle programs centered on the family cancer experience, we utilized an existing PC family registry. Our results may not replicate in families with other cancers. In addition, although we employed analytic strategies (i.e., GEE) to account for possible non-independence in the data, our results should be interpreted with caution. Finally, our sample was very homogeneous, primarily white, limiting our ability to generalize the findings to other racial or ethnic groups who are not spared the risk of PC and other cancers. Future research should be conducted with other ethnic groups and in other countries to explore how cultural differences may impact interest levels in lifestyle interventions aimed at reducing cancer risk. Strengths of this study include the use of validated measures, the large sample size, and the use of an existing family registry which made our study cost-effective and feasible.

## Conclusion

In conclusion, our data suggest that family-based cancer risk reduction programs may be a promising addition to cancer prevention efforts. Utilizing newsletters or Web/Internet-based technology will expand the reach of such programs and may be more cost-effective than in-person interventions, although each of these must be carefully evaluated. Our findings provide important preliminary data that investigators can use to translate lifestyle research to the cancer survivor communities.

## Competing interests

The author(s) declare that they have no competing interests.

## Authors’ contributions

LAH, PSS, CAP, and GMP conceived of the study, participated in the study design, and constructed the survey. PSS and TAB coordinated the research efforts and data collection. PAD, AN, and KGR performed the statistical analysis. LAH, PSS, TAB, CAP, SLE, CRB and GMP made contributions to interpretation of the data and drafted of the manuscript. All authors read and approved the final manuscript.
